# Synthesis, Modelling, and Anticonvulsant Studies of New Quinazolines Showing Three Highly Active Compounds with Low Toxicity and High Affinity to the GABA-A Receptor

**DOI:** 10.3390/molecules22020188

**Published:** 2017-01-24

**Authors:** Mohamed F. Zayed, Saleh K. Ihmaid, Hany E. A. Ahmed, Khaled El-Adl, Ahmed M. Asiri, Abdelsattar M. Omar

**Affiliations:** 1 Department of Pharmacognosy and Pharmaceutical Chemistry, College of Pharmacy, Taibah University, Al-Madinah, Al-Munawarah 41477, Saudi Arabia; sihmaid@taibahu.edu.sa (S.K.I.); heahmad@taibahu.edu.sa (H.E.A.A.); amasiri@taibahu.edu.sa (A.M.A.); 2 Department of Pharmaceutical Chemistry, Faculty of Pharmacy, Al-Azhar University, Cairo 11884, Egypt; eladlkhaled74@yahoo.com (K.E.-A.); asmansour@kau.edu.sa (A.M.O.); 3 Pharmaceutical Organic Chemistry Department, Faculty of Pharmacy, Al-Azhar University, Cairo 11884, Egypt; 4 Department of Pharmaceutical Chemistry, Faculty of Pharmacy, King Abdulaziz University, Jeddah 21589, Saudi Arabia

**Keywords:** design, synthesis, anticonvulsant, quinazoline, epilepsy, neurotoxicity

## Abstract

Some novel fluorinated quinazolines (**5a**–**j**) were designed and synthesized to be evaluated for their anticonvulsant activity and their neurotoxicity. Structures of all newly synthesized compounds were confirmed by their infrared (IR), mass spectrometry (MS) spectra, ^1^H nuclear magnetic resonance (NMR), ^13^C-NMR, and elemental analysis (CHN). The anticonvulsant activity was evaluated by a subcutaneous pentylenetetrazole (scPTZ) test and maximal electroshock (MES)-induced seizure test, while neurotoxicity was evaluated by a rotorod test. The molecular docking was performed for all newly-synthesized compounds to assess their binding affinities to the GABA-A receptor in order to rationalize their anticonvulsant activities in a qualitative way. The data obtained from the molecular modeling was correlated with that obtained from the biological screening. These data showed considerable anticonvulsant activity for all newly-synthesized compounds. Compounds **5b**, **5c**, and **5d** showed the highest binding affinities toward the GABA-A receptor, along with the highest anticonvulsant activities in experimental mice. These compounds also showed low neurotoxicity and low toxicity in the median lethal dose test compared to the reference drugs. A GABA enzymatic assay was performed for these highly active compounds to confirm the obtained results and explain the possible mechanism for anticonvulsant action. The most active compounds might be used as leads for future modification and optimization.

## 1. Introduction

Epilepsy is a neurological disorder characterized by recurrent seizures [1,2,3,4,5]. It represents a major health problem and inflicts millions of people worldwide [6,7,8,9,10]. Approximately, one third of epilepsy patients have a multidrug resistance (MDR) syndrome and refractory epilepsy (RE). The processes of (MDR) and (RE) result from enhanced expression and up-regulation of p-glycoprotein (P-GP) into the brain. This leads to defective access of the anticonvulsant drugs to their target in the central nervous system (CNS) [8].

Despite the non-stop discovery of new antiepileptic drugs (AEDs), the treatment process is still inadequate and unsatisfactory due to several side effects, such as neurotoxicity, depression, drowsiness, ataxia, gastrointestinal disturbances, megaloblastic anemia, hirsutism, and other CNS disorders [5]. 

**Figure 1:**
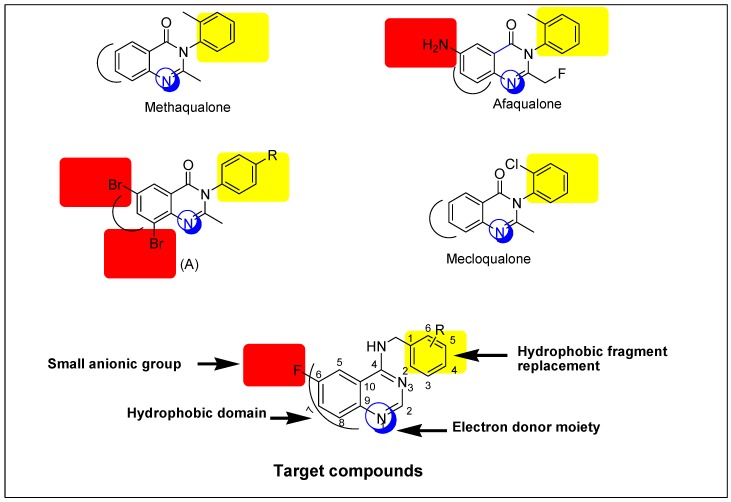
Structural similarities and pharmacophoric features of reported and designed quinazolines (**5a**–**j**) as anticonvulsant agents.

The quinazoline ring represents a very important advantaged substructure in organic synthesis for preparing different biologically active molecules [4,5,6]. Typically, the most important pharmacological effects that have been reported for quinazoline derivatives are sedative-hypnotic and anticonvulsant activities [9]. Many quinazoline derivatives were reported as anticonvulsants but none of these derivatives are clinically approved because these derivatives had high neurotoxicity values (TD_50_) and low protective index values (PI) [5]. Therefore, we need to search for safer and more effective anticonvulsants. Based on that, some novel quinazoline derivatives were designed and prepared to be biologically tested as anticonvulsants. The general structure of target compounds is shown in Figure 1.

A literature study explains that the existence of quinazoline nucleus in the molecule is a necessary requirement for the anticonvulsant activity. This nucleus can be substituted on the heteroatom or the aromatic ring, as shown in Figure 1. Moreover, the quinazoline moiety with a suitable substituent, mostly amine or a substituted amine at the 4-position, and either a halogen or electron-rich substituent at the 6-position or 8-position, is known to improve antiepileptic activity [5,6]. Based on the previous rationale, it was believed to be valuable to study the effects of two pharmacophoric groups, like quinazoline and aromatic amine, in one molecule for anticonvulsant activity. The quinazoline nucleus is attached to an aromatic unit via a (CH_2_) linker. This design formed new quinazoline derivatives that possess essential features of the lead structure methaqualone. Selection of the aromatic amines was following the Hansch manual method of drug design which was proposed by Topliss [11]. It is a technique in which the substituents are nominated, ordered, and tested based on the effectiveness. A fluorine atom was selected as a substituent on the quinazoline moiety owing to its safety, small size, low steric interference, and ready availability [12,13]. Additionally, the presence of fluorine at the 6-position could enhance the pharmacokinetic and pharmacodynamic properties of the newly-synthesized compounds [6,13]. The title compounds were synthesized as an attempt to find new efficient and safe anticonvulsants. Similarities between the reported anticonvulsants methaqualone, afaqualone, mecloqualone, compound (**A**) and our newly-designed compounds are shown in Figure 1.

## 2. Results and Discussion

### 2.1. Chemistry

**Scheme 1:**
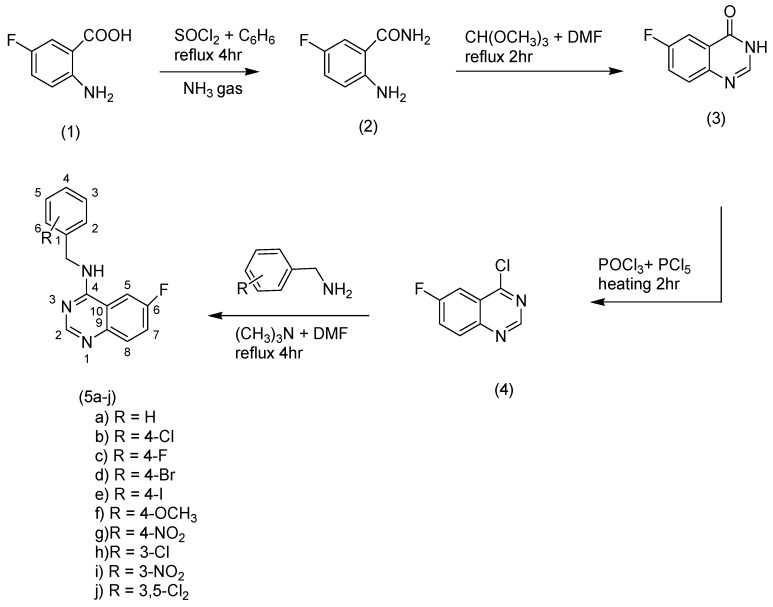
Synthesis of the target compounds (**5a**–**j**).

Substituted-6-fluoro-quinazoline-4-amine (**5a**–**j**) derivatives were synthesized according to the methods outlined in Scheme 1. Synthesis of 2-amino-5-fluorobenzamide (**2**) was accomplished by refluxing of thionylchloride and 2-amino-5-fluorobenzoic acid in benzene, then the mixture was exposed to dry ammonia gas. 6-fluroquinazolin-4(3*H*)-one (**3**) was prepared by reaction of the amide compound (**2**) with trimethoxymethane in dimethylformamide (DMF). Chlorination of compound (**3**) was performed by heating (**3**) with a mixture of phosphorous oxychloride and phosphorous pentachloride. A nucleophilic displacement reaction for the chloroquinazoline (**4**) was accomplished through treating of compound (**4**) with different substituted aromatic amines in the presence of trimethylamine to obtain the title compounds (**5a**–**j**). Infrared, mass spectrometry, ^1^H nuclear magnetic resonance (NMR), ^13^C-NMR, and elemental analyses were performed to confirm all structures. The title compounds contained different substituents with different electronic environments, as shown in Scheme 1. These substituents are joined to the fixed moiety 6-fluroquinazoline. They included different hydrophilic and hydrophobic groups, either electron-deficient or electron-rich groups, to study and compare their anticonvulsant activity.

**Figure 2:**
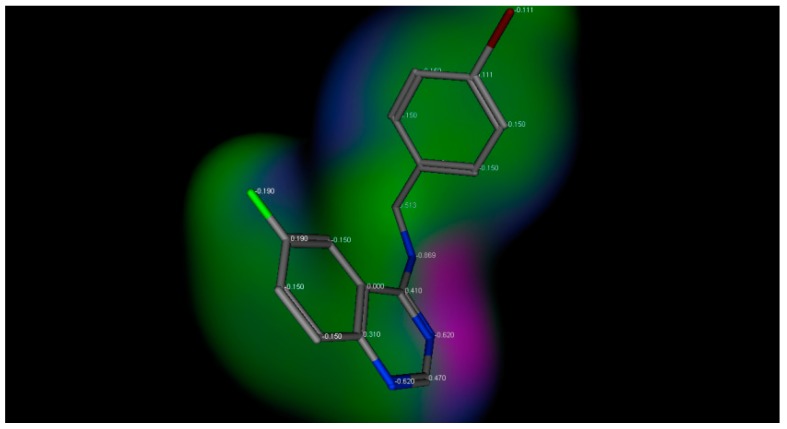
Electronic environment of the title compounds exemplified by compound **3d.** Green color indicates hydrophobic area (low electron density), pink color indicates the hydrogen bond area (high electron density), and the blue color indicates the mild polar area (mild electron density).

The title compounds contain different substituents with different electronic environments, as shown in Figure 2. These substituents are joined to the fixed moiety 6-fluroquinazoline. They included different hydrophilic and hydrophobic groups, either electron-deficient or electron-rich groups, to study and compare their anticonvulsant activity. Figure 2 shows the electronic environment of the title compounds represented by compound **3d**.

### 2.2. Anticonvulsant Screening 

The anticonvulsant screening of the title compounds was performed according to standard procedures [14,15,16]. The initial screening was performed at 0.5 mmol/kg of all synthesized compounds (**5a**–**j**) by chemical induction of the convulsions using pentylenetetrazole (PTZ) and electrical induction of convulsions using maximal electroshock (MES) models of seizures [17,18]. The scPTZ model has been reported to be a good predictor of those anticonvulsants to protect against generalized spike-wave epilepsies which are known as absence seizures [19,20]. These anticonvulsants are functionally related to methaqualone. The MES model is used to predict the ability of anticonvulsants to protect against generalized tonic-clonic seizures which are functionally similar to phenytoin [5,6]. Thus, scPTZ and MES screenings became the best broadly applicable seizure models for preliminary detection of antiepileptic agents.

The primary anticonvulsant screening for the newly synthesized compounds displayed that all of them were active in PTZ test at 0.5 mmol/kg. Compounds **5b**, **5c**, **5d**, and **5e** showed 100% protection, compounds **5a** and **5f** showed 83% protection, while compounds **5g**, **5h**, and **5j** showed 66% protection. For the MES test, compound **5f** showed 66% protection, compounds **5a** and **5g** showed 50% protection, while compounds **5h**, **5i**, and **5j** showed 33% protection. These results are elucidated in Table 1.

The most active compounds **5b**, **5c**, and **5d**, were exposed to further investigations in mice to determine and quantify their anticonvulsant activity. Further investigations included the study of the effective dose that caused protection for 50% of mice (ED_50_) and that produce minimal neurological toxicity in 50% of mice (TD_50_) to determine their neurotoxicity, as shown in Table 2.

Compounds **5b**, **5c**, and **5d** had (ED_50_) values of 152, 165, and 140 mg/kg, respectively (see Table 2). The reference drugs methaqualone and valproate produced (ED_50_) values of 200 and 300 mg/kg, respectively. The ED_50_ values of the test compounds were smaller than the reference drugs at molar doses. The protective index (PI = TD_50_/ED_50_) is an index representing the margin of safety between anticonvulsant doses and doses of anticonvulsant drugs producing acute adverse effects, like motor coordination impairment, ataxia, or any other neurotoxic symptoms [9,10]. The highest active compounds **5b**, **5c**, and **5d**, had TD_50_ values of 270, 350, and 320 mg/kg. These values revealed that these compounds exerted low neurological disorders. From the results of ED_50_ and TD_50_, compounds **5b**, **5c**, and **5d** had PI values of 1.78, 2.12, and 2.28, while methaqualone and valproate had PI values of 2 and 1.5. These results showed a high safety margin of the title compounds compared to the reference drugs. The therapeutic index (TI = LD_50_/ED_50_) is a comparison of the amount of a therapeutic agent that causes the therapeutic effect to the amount that causes death in mice [5,9]. Compounds **5b**, **5c**, and **5d** had LD_50_ values of 580, 430, and 550 mg/kg, and TI values of 3.82, 2.6, and 3.93, while methaqualone and valproate had TI values of 2.5 and 1.67. 

**Figure 3:**
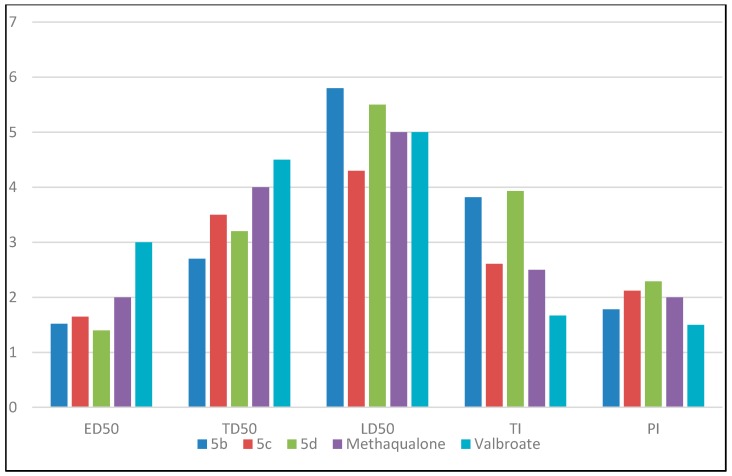
Comparison between ED_50_, TD_50_, LD_50_, TI, and PI for the highest active compounds **5b**, **5c** and **5d**. The values of ED_50_, TD_50_, are LD_50_ are divided by 100 for simplicity.

Figure 2 represents a comparison between the values of ED_50_, TD_50_, LD_50_, TI, and PI for the highest active compounds **5b**, **5c**, and **5d**, against reference drugs methaqualone and valproate. The results of the anticonvulsant screening explained the broad spectrum activity of the title compounds against partial and generalized epilepsy. Structure activity relationship (SAR) studies explained that different substitutions on the aromatic ring of the aromatic amine exerted varied anticonvulsant activity. A significant variation in the anticonvulsant activity was obtained by changing the electronic nature and the position of the substituent group attached to the aromatic ring. Based on the data shown above, all para-substituted analogs had higher activity than meta- or di-substituted analogs. The electron withdrawing groups attached to the aromatic ring at the para position produced more anticonvulsant activity than that containing electron-donating groups or remained unsubstituted. For example, electronic withdrawing groups, like bromo substitution at the para position from the aromatic ring, improved the anticonvulsant activity, while other derivatives showed less activity. Compounds activity was arranged as the following: 4Br (**5d**) > 4Cl (**5b**) > 4F (**5c**) > 4I (**5e**) > 4OCH_3_ (**5f**) > H (**5a**) > 4NO_2_ (**5g**) > 3Cl (**5h**) > 3,5Cl_2_ (**5j**) > 3NO_2_ (**5i**). A comparison between ED_50_, TD_50_, LD_50_, TI, and PI for the highest active compounds **5b**, **5c**, and **5d**, is shown in Figure 3. 

### 2.3. Docking Studies

Many quinazoline derivatives were reported as GABA-A receptor stimulants [21,22]. The GABA-A receptor is an ionotropic receptor and a ligand-gated ion channel. Its endogenous ligand is γ-aminobutyric acid (GABA), the major inhibitory neurotransmitter in the central nervous system. Upon activation, the GABA-A receptor selectively conducts Cl^−^ through its pore, resulting in hyperpolarization of the neuron. This causes an inhibitory effect on neurotransmission by diminishing the chance of a successful action potential [21]. The active site of the GABA-A receptor is the binding site for GABA and several drugs, such as muscimol. The protein also contains a number of different allosteric binding sites which modulate the activity of the receptor indirectly. These allosteric sites are the targets of various other drugs, including benzodiazepines, barbiturates, and ethanol [23,24]. Methaqualone is a positive allosteric GABA-A receptor modulator. It binds to allosteric sites on the GABA-A receptor complex and affects it in a positive manner, causing increased efficiency of the main site and, therefore, an indirect increase in Cl^−^ conductance [23,24].

Our target compounds were designed to replace the carbonyl group (hydrogen bond acceptor) at the 4-position of methaqualone by an amino group (hydrogen bond donor), which occupy the same position at the active site. On the other hand, this amino group was joined to a distal-substituted phenyl moiety through a CH_2_ linker which imparts a good hydrophobic domain which occupies the same position at the active site occupied by 2-chlorophenyl at the 3-position of methaqualone. The phenyl group of methaqualone was substituted at the 2-position while, in our designed compounds, it was substituted at different positions. All derivatives were substituted with fluorine atoms to increase hydrophobic interactions. These modifications were made in order to increase hydrogen bonding at the 4-position and also increase hydrophobic bonding to study their effects on SAR, hoping to obtain new compounds with higher GABA affinity and consequently higher anticonvulsant activity.

In order to qualify the docking results in terms of accuracy of the predicted binding conformations in comparison with the experimental procedure, the reported GABA-A receptor stimulant drug (Methaqualone) was used as a reference ligand. The docking study has been conducted to predict the binding mode and to rationalize the observed biological activity. 

**Figure 4:**
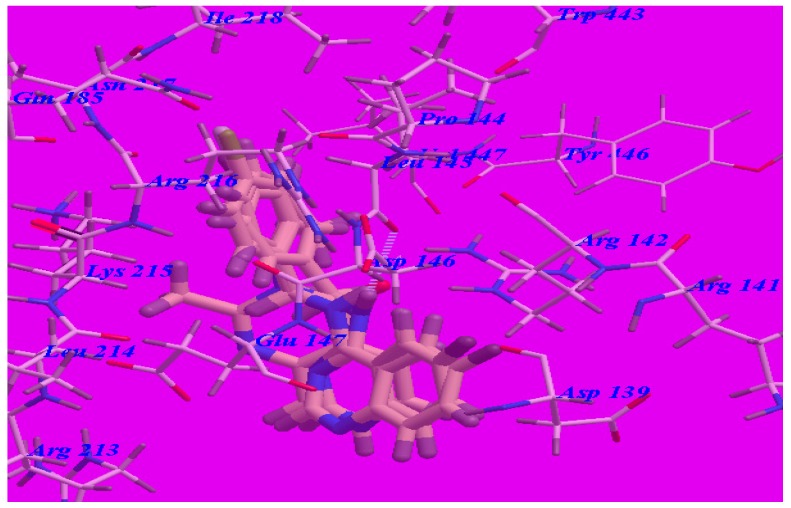
Docking and superimposition of compounds **5d**, **5b**, **5c**, and methaqualone in the vicinity of the GABA-A receptor (4COF).

The obtained results indicated that all studied ligands have similar position and orientation inside the putative binding site of GABA-A receptor (PDB code 4COF) which reveals a large space bounded by a membrane-binding domain that serves as an entry channel for substrate to the active site (see Figure 4). In addition, the affinity of any small molecule can be considered as a unique tool in the field of drug design. There is a relationship between the affinity of organic molecules and the free energy of binding. This relationship can contribute in prediction and interpretation of the activity of the organic compounds toward the specific target protein [12,15]. The obtained results of the free energy of binding (∆G) explained that most of these compounds had good binding affinity toward the receptor and the computed values reflected the overall trend (see Table 3). 

**Figure 5:**
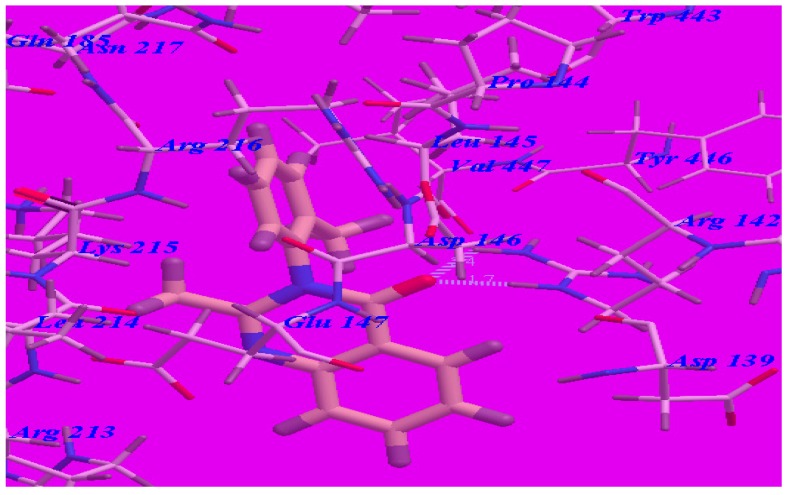
Predicted binding mode for methaqualone with the GABA-A receptor (4COF). H-bonds are indicated by dotted lines.

The proposed binding mode of methaqualone revealed an affinity value of –50.69 kcal/mol and the carbonyl group formed two H-bonds with *Arg142* (–NH groups) with distances of 1.65 Å and 2.38 Å (see Figure 5). The quinazolinone moiety occupied the hydrophobic pocket formed by *Asp139*, *Arg142*, *Asp146*, *Glu147*, and *Arg213*. The phenyl ring at the 3-position was oriented in the hydrophobic cleft formed by *Leu145*, *Asp146*, *Lys215*, *Arg216*, and *Ile218*. The methyl group at 2-position was oriented in the hydrophobic cleft formed by *Arg142*, *Leu145*, and *Val447*.

**Figure 6:**
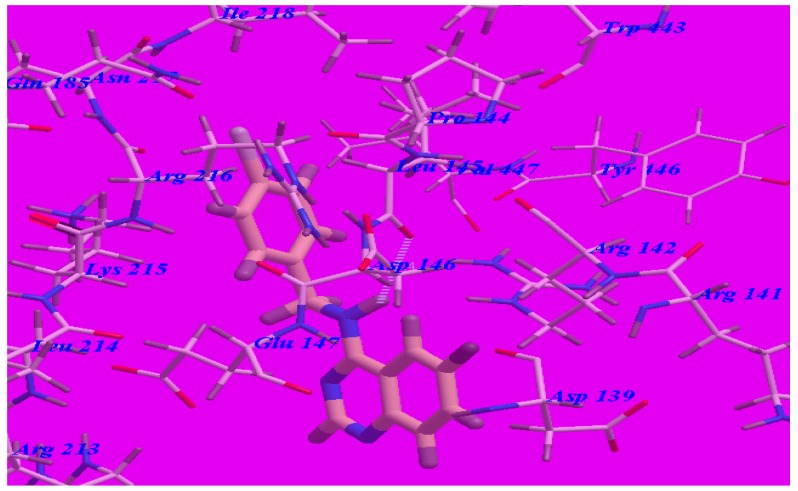
Predicted binding mode for compound **5d** with a GABA-A receptor.

The proposed binding mode of compound **5d** (affinity value of –69.68 kcal/mol and one H-bond) (see Figure 6) is virtually the same as that of methaqualone, where the NH group at the 4-position formed a hydrogen bond with *Leu145* (–O atom) with a distance of 2.43 Å. The 6-fluoroquinazoline moiety occupied the hydrophobic pocket formed by *Asp139*, *Arg141*, *Arg142*, *Asp146*, *Glu147*, and *Arg213*. The distal phenyl ring was oriented in the hydrophobic cleft formed by *Leu145, Asp146*, *Glu147*, *Lys215*, *Arg216*, and *Ile218*. These interactions of compound **5d** may explain the highest binding free energy and anticonvulsant activity.

**Figure 7:**
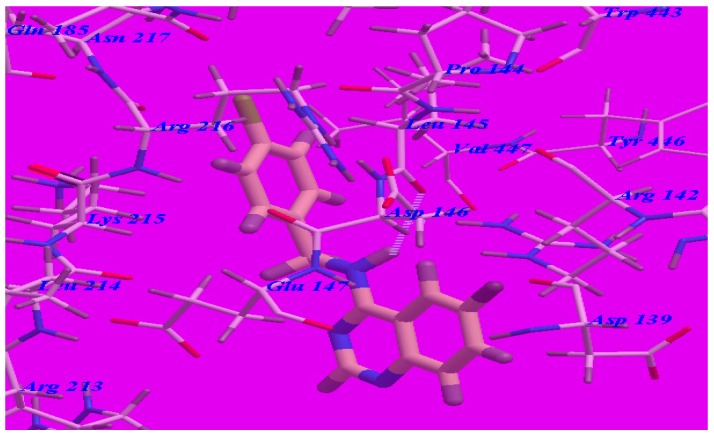
Predicted binding mode for compound **5b** with a GABA-A receptor.

**Figure 8:**
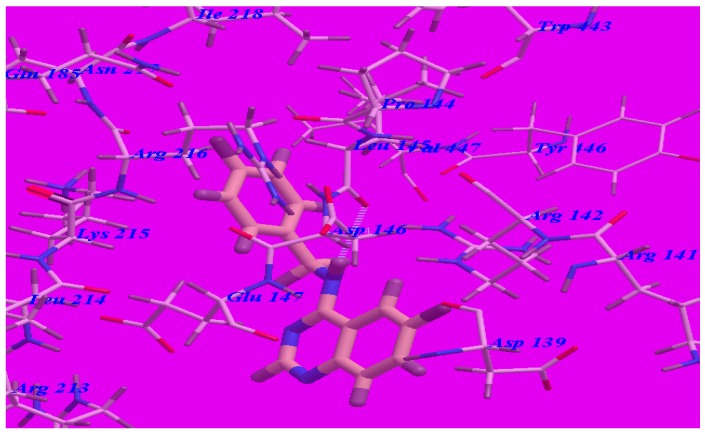
Predicted binding mode for compounds **5c** with a GABA-A receptor.

Furthermore, the proposed binding modes of compounds **5b** (affinity value of −68.97 kcal/mol and one H-bond) (see Figure 7) and **5c** (affinity value of −67.50 kcal/mol and one H-bond) (see Figure 8) are virtually the same as that of **5d** where the three derivatives were superimposed. These interactions may explain the high binding free energy and anticonvulsant activity. 

### 2.4. GABA Enzymatic Assay

**Figure 9:**
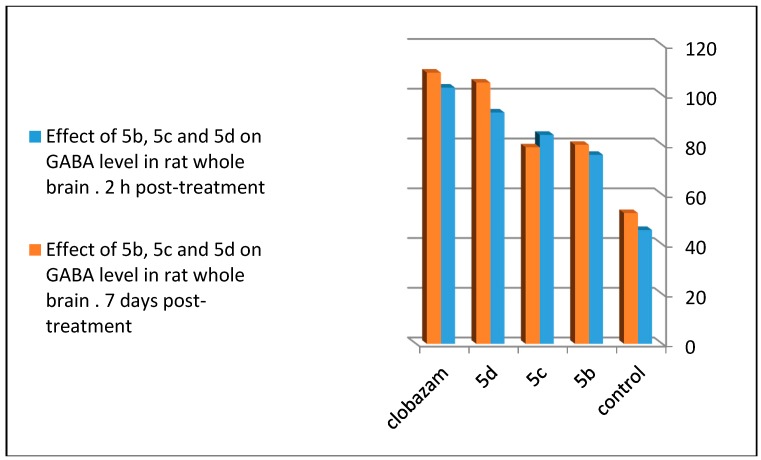
Comparison between the effects of **5b**, **5c**, and **5d** on the GABA level in rat whole brain 2 h post-treatment and seven days post-treatment.

To follow the effect on the GABA receptor, **5b**, **5c**, and **5d** were subjected to neurochemical examination for the determination of the GABA level in different areas of whole rat brain. The three tested compounds statistically increased the GABA concentration compared to the control with **5d** (*p* < 0.001 level of significance), and **5b** and **5c** (*p* < 0.004, significant after 2 h of administration). Chronic oral administration for seven days showed a non-significant result for **5d** compared to **5b** and **5c** (*p* < 0.01 significant). Tested compounds revealed lesser or closely equal concentrations of GABA to clobazam, which was used as the standard anticonvulsant drug [16] (Table 4, Figure 9). From these result, one can conclude that the para bromo-substituted quinazoline ring in **5d**, para chloro-substituted quinazoline ring in **5b**, and para fluoro-substituted quinazoline ring in **5c** increased the effectiveness of the compound at the GABA receptor through increasing its level of concentration in the rat brain assay.

## 3. Materials and Methods

### 3.1. General

Chemicals were obtained from Sigma-Aldrich (Aldrich chemical Co., Milwaukee, WI, USA). A Griffin melting point apparatus (Griffin, Valdosta, GA, USA) was used to measure melting points (m.p.). A Nicolet IR 300 (Thermo Fisher Scientific, Barrington, RI, USA) was used to measure the infrared spectra. ^1^H**-**NMR and ^13^C**-**NMR (Varian, Palo Alto, CA, USA) were performed in deuterated dimethyl sulfoxide (DMSO-*d*_6_) using a Varian 300 MHz instrument. Tetramethylsilane (TMS) was used as internal standard. MS spectra were measured using a JEOL-SX-102 electron impact ionization instrument (Joel, Peabody, MA, USA). Evaporation processes were performed under reduced pressure by using a Buchi rotary evaporator (Buchi, Postfach, Flawil, Switzerland). Elemental analyses (C, H, N) were accomplished using a Perkin-Elmer 240C analyzer (Perkin-Elmer, Norwalk, CT, USA). The synthesized compounds were within ±0.4% of the theoretical values. Purity of the newly-synthesized compounds was above 99.6%.

### 3.2. General Method of the Synthesis of 2-Amino-5-Fluorobenzamide *(**2**)*

A suspension of 2-amino-5-fluorobenzoic acid (1) (1.0 g, 6.3 mmol) in benzene (30 mL) under reflux was mixed with thionyl chloride (1.5 g, 12.6 mmol). The resulting mixture was stirred under reflux for 4 h, and then evaporated. The residue was dissolved in 200 mL of benzene and treated with anhydrous ammonia gas at room temperature. After removing the solvent, compound **2** was crystalized from ethanol and obtained as brown powder [25]. Yield 75%; m.p.: 144–146 °C; ^1^H-NMR (DMSO-*d*_6_, 300 MHz) δ: 5.72(s, 2H, NH_2_,), 6.54(s, CONH_2_, 2H) 7.63 (d, 1H, *J* = 8.6 Hz, Ar-H), 7.92 (d, 1H, *J* = 7.4 Hz, Ar-H), 8.14 (s, 1H, Ar-H). C_7_H_7_FN_2_O (154.05): MS (ESI) *m*/*z* 155.05 [M + 1].

### 3.3. General Method of the Synthesis of 6-Fluroquinazolin-4(3H)-one *(**3**)*

This compound was previously reported [25] but we used another method for preparation of this compound to improve the yield. In a 10 mL volume stainless steel pressure-resistant vessel were placed (1.54 g, 0.01 mol) of 2-amino-5-fluorobenzamide **2**, (1.06 g, 0.01 mol) of trimethoxymethane, and 5 mL of DMF. The reaction was heated at 170 °C for 2 h. After the reaction was completed the product was cooled, collected, and crystallized from ethyl alcohol. Yield 70%; mp: 252–254 °C; ^1^H-NMR (DMSO-*d*_6_, 300 MHz) δ: 7.67 (d, 1H, *J* = 8.6 Hz, ArH), 7.85 (d, 1H, *J* = 9.7 Hz, ArH), 8.12 (s, 1H, ArH), 8.73 (dd, 1H, *J* = 8.4 Hz and 4.8 Hz, ArH), 11.83(s, 1H, NH), C_8_H_5_FN_2_O (164.04): MS (ESI) *m*/*z* 165.04 [M + 1].

### 3.4. General Method of the Synthesis of 4-Chloro-6-Fluoro-Quinazoline *(**4**)*

A mixture of compound **3** (1.64 g, 0.01 mol) in phosphorus oxychloride (0.05 mol, 7.5 mL) and phosphorous pentachloride (0.03 mol, 6 g) was heated on a water bath for 2 hr. The reaction mixture was cooled and poured slowly onto crushed ice. The resulted precipitate was filtered off, washed with water, dried, and purified by column chromatography with hexanes/ethyl acetate to afford the desired product. Yield 65%; mp: 140–142 °C; ^1^H-NMR (DMSO-*d*_6_, 300 MHz) δ 8.12 (s, 1H, ArH), 7.47 (dd, 1H, *J* = 7.9 Hz and 2.8 Hz, ArH), 7.53 (dd, 1H, *J* = 8.6 Hz and 5.1 Hz, ArH), 7.62 (s, 1H, ArH); C_8_H_4_ClFN_2_ (182): MS (ESI) *m*/*z* 183 [M + 1]

### 3.5. General Method of the Synthesis of Quinazoline Derivatives *(**5a**–**j**)*

Compound **4** (1.84 g, 0.01 mol) was mixed with (1.06 g, 0.01 mol) of trimethylamine and (0.01 mol) of the appropriate aromatic amine in 5 mL of dimethylformamide (DMF). The mixture was refluxed for 4 h, cooled, and poured on crushed ice. The crystals were collected and crystallized from ethyl alcohol. The melting points for all synthesized compounds were above 300 °C.

*N-Benzyl-6-fluoroquinazoline-4-amine* (**5a**): Yield 68%; white solid in a purity higher than 98%, ^1^H-NMR (DMSO-*d*_6_): δ 3.95 (s, 2H, CH_2_), 5.12 (s, 1H, NH), 6.21–7.93 (m, 9H, Ar-H); ^13^C-NMR (DMSO-*d*_6_): δ 48.57, 57.18, 112.48, 114.16, 124.3, 125, 126.22, 127.14, 128.82, 128.84, 135.21, 136, 140.37, 153.4, 162.3. MS (ESI) *m*/*z* 254.1 [M + 1]. Anal. Calcd. For C_15_H_12_FN_3_ (253.1): C, 71.13; H, 4.78; N, 16.59. Found C, 71.34; H, 5.01; N, 16.54.

*N-(4-Chlorobenzyl)-6-fluoroquinazoline-4-amine* (**5b**): Yield 65%; white solid in a purity higher than 98%, ^1^H-NMR (DMSO-*d*_6_): δ 3.93 (s, 2H, CH_2_), 5.34 (s, 1H, NH), 6.31–7.73 (m, 8H, Ar-H); ^13^C-NMR (DMSO-*d*_6_): δ 47.39, 57.78, 111.2, 113.86, 122.93, 124.14, 125.72, 127.34, 128, 128.87, 135, 136.21, 140.87, 151.24, 161.43. MS (ESI) *m*/*z* 288.06 [M + 1]. Anal. Calcd. For C_15_H_11_ClFN_3_ (287.06): C, 62.62; H, 3.85; N, 14.60. Found C, 62.33; H, 4.01; N, 14.53.

*N-(4-Fluorobenzyl)-6-Fluoroquinazoline-4-Amine* (**5c**): Yield 62%; white solid in a purity higher than 99%, ^1^H-NMR (DMSO-*d*_6_): δ 3.87 (s, 2H, CH_2_), 5.29 (s, 1H, NH), 6.52-7.82 (m, 8H, Ar-H); ^13^C-NMR (DMSO-*d*_6_): δ 46.89, 57.41, 112.32, 114.16, 121.97, 122.26, 125.12, 126.74, 127.45, 128.52, 134.37, 136, 139.87, 151.36, 162.13. MS (ESI) *m*/*z* 272.09 [M + 1]. Anal. Calcd. For C_15_H_11_F_2_N_3_ (271.09): C, 66.42; H, 4.09; N, 15.49. Found C, 66.23; H, 4.12; N, 15.51.

*N-(4-Bromobenzyl)-6-fluoroquinazoline-4-amine* (**5d**): Yield 65%; white solid in a purity higher than 97%, ^1^H-NMR (DMSO-*d*_6_): δ 3.79 (s, 2H, CH_2_), 5.41 (s, 1H, NH), 6.64-7.91 (m, 8H, Ar-H); ^13^C-NMR (DMSO-*d*_6_): δ 44.19, 55.81, 111.62, 114.86, 120.87, 122.52, 124.32, 126, 128.25, 131.24, 135.27, 136.81, 138.27, 152.46, 160.73. MS (ESI) *m*/*z* 332.01 [M + 1]. Anal. Calcd. For C_15_H_11_BrFN_3_ (331.01): C, 54.24; H, 3.34; N, 12.65. Found C, 54.43; H, 3.52; N, 12.41.

*N-(4-Iodobenzyl)-6-fluoroquinazoline-4-amine* (**5e**): Yield 67%; white solid in a purity higher than 96%, ^1^H-NMR (DMSO-*d*_6_): δ 3.89 (s, 2H, CH_2_), 5.41 (s, 1H, NH), 6.64–7.91 (m, 8H, Ar-H); ^13^C-NMR (DMSO-*d*_6_): δ 44.28, 54.92, 110.52, 112, 120.17, 122.47, 123.52, 125.47, 127.15, 130.21, 136.37, 137.71, 138.52, 152, 160.24. MS (ESI) *m*/*z* 380 [M + 1]. Anal. Calcd. For C_15_H_11_FIN_3_ (379): C, 47.51; H, 2.92; N, 11.08. Found C, 47.41; H, 3.22; N, 11.21.

*N-(4-Methoxybenzyl)-6-fluoroquinazoline-4-amine* (**5f**): Yield 60%; white solid in a purity higher than 98%, ^1^H-NMR (DMSO-*d*_6_): δ 3.81 (s, 2H, CH_2_), 4.05 (s, 3H, OCH_3_), 5.36 (s, 1H, NH), 6.57–7.79 (m, 8H, Ar-H); ^13^C-NMR (DMSO-*d*_6_): δ 45, 56.12, 58.72, 112.62, 114.31, 118.27, 122.47, 122.72, 125.17, 128.18, 131.61, 137.47, 139.71, 142.52, 152.21, 163.34. MS (ESI) *m*/*z* 284.11 [M + 1]. Anal. Calcd. For C_16_H_14_FN_3_O (283.11): C, 67.83; H, 4.98; N, 14.83. Found C, 67.75; H, 5.23; N, 14.91.

*N-(4-Nitrobenzyl)-6-fluoroquinazoline-4-amine* (**5g**): Yield 62%; white solid in a purity higher than 97%, ^1^H-NMR (DMSO-*d*_6_): δ 3.91 (s, 2H, CH_2_), 5.41 (s, 1H, NH), 6.82–8.11 (m, 8H, Ar-H); ^13^C-NMR (DMSO-*d*_6_): δ 42.58, 56.72, 111.72, 114.61, 118.57, 122, 123.52, 125.47, 127.15, 130.21, 136.37, 137.71, 138.52, 152, 161.54. MS (ESI) *m*/*z* 299.08 [M + 1]. Anal. Calcd. For C_15_H_11_FN_4_O_2_ (298.08): C, 60.40; H, 3.72; N, 18.78. Found C, 60.37; H, 4.01; N, 18.65.

*N-(3-Chlorobenzyl)-6-fluoroquinazoline-4-amine* (**5h**): Yield 65%; white solid in a purity higher than 98%, ^1^H-NMR (DMSO-*d*_6_): δ 3.94 (s, 2H, CH_2_), 5.61 (s, 1H, NH), 6.64–7.94 (m, 8H, Ar-H); ^13^C-NMR (DMSO-*d*_6_): δ 40.28, 54.78, 114.12, 116.92, 118.53, 120, 122.12, 126.57, 128.21, 131.41, 136.64, 137.01, 139.42, 152, 160.54. MS (ESI) *m*/*z* 288.06 [M + 1]. Anal. Calcd. For C_15_H_11_ClFN_3_ (287.06): C, 62.62; H, 3.85; N, 14.60. Found C, 62.51; H, 3.96; N, 14.43.

*N-(3-Nitrobenzyl)-6-fluoroquinazoline-4-amine* (**5i**): Yield 62%; white solid in a purity higher than 98%, ^1^H-NMR (DMSO-*d*_6_): δ 3.89 (s, 2H, CH_2_), 5.31 (s, 1H, NH), 6.74–8.21 (m, 8H, Ar-H); ^13^C-NMR (DMSO-*d*_6_): δ 42.76, 54.82, 111.82, 114.91, 116.78, 122.32, 123, 124.62, 127.81, 132.41, 136.57, 138.32, 141.42, 150.6, 162.31. MS (ESI) *m*/*z* 299.09 [M + 1]. Anal. Calcd. For C_15_H_11_FN_4_O_2_ (298.09): C, 60.40; H, 3.72; N, 18.78. Found C, 60.21; H, 3.61; N, 18.95.

*N-(3,5-Dichlorobenzyl)-6-fluoroquinazoline-4-amine* (**5j**): Yield 67%; white solid in a purity higher than 97%, ^1^H-NMR (DMSO-*d*_6_): δ 3.91 (s, 1H, NH), 5.48 (s, 2H, 2NH), 6.74–7.97 (m, 7H, Ar-H); ^13^C-NMR (DMSO-*d*_6_): δ 40.63, 52.78, 112.98, 116, 116.93, 121.24, 122.76, 124.85, 128.71, 130.61, 134.84, 138.21, 140.02, 152.41, 160.04. MS (ESI) *m*/*z* 322.02 [M + 1]. Anal. Calcd. For C_15_H_10_Cl_2_FN_3_ (321.02): C, 55.92; H, 3.13; N, 13.04. Found C, 56.07; H, 3.31; N, 13.22.

### 3.6. Docking Studies

In the present work, all the target compounds were subjected to docking study to explore their binding mode to the GABA-A receptor. All modeling experiments were performed using the molsoft program which provides a unique set of tools for the modeling of protein/ligand interactions. It predicts how small flexible molecules, such as substrates or drug candidates, bind to a protein of a known 3D structure represented by grid interaction potentials. Each experiment used the biological target GABA-A receptor downloaded from the Brookhaven Protein Databank (PDB) (http://www.rcsb.org/pdb/explore/explore.do?structureId=4COF) [26]. 

### 3.7. Pharmacological Tests

Swiss albino mice 25–40 g (12–15 weeks old) were used in the experiments. These mice were obtained from the Animal Centre, King Abdul-Aziz University. The mice were kept under typical conditions of humidity, temperature (23 ± 2 °C), and light (12 h light/12 h dark). The animals were fed with a standard mouse pellet diet and had free access to water. The protocol used for the testing of animals was approved by the Institutional Animal Ethics Committee (approval number 6063/1435). All of the biological experiments were carried out according to the respective internationally valid guidelines. The test group and vehicle control group included six animals. Median toxic dose (TD_50_) is the dose required to get 50% of the animals reporting specific toxic effects. It was determined by a rotorod test in mice [5,9]. Protective index is the ratio between TD_50_ and ED_50_. This was determined from the equation PI = TD_50_/ED_50_. The median lethal dose (LD_50_) is the amount of therapeutic agent that leads to 50% death in mice. LD_50_ was calculated from the dose-response curves with at least four doses [9]. Therapeutic index (TI) is the ratio between the dosage of a drug that causes a lethal effect and the dosage that causes a therapeutic effect. It was determined from the equation TI = LD_50_/ED_50_ [5].

### 3.8. Anticonvulsant Screening

Anticonvulsant screening was evaluated by the pentylentetrazole (PTZ) model and maximal electroshock (MES) model according to the reported procedures [20]. Test compounds were dissolved in 10% DMSO and injected intraperitoneal (i.p.) at a dose of 0.5 mmol/kg one half hour before seizure induction. Sodium valproate (1.8 mmol/kg) and methaqualone (0.8 mmol/kg) were used as two reference drugs. Seizure induction in the MES test was obtained by an electrical stimulus (50 mA, 60 Hz) from an electroconvulsiometer apparatus. The electrical current was applied for 0.2 s via euricular electrodes. Protection against these seizures was identified by the abolition of the hind leg and tonic maximal extension component of the seizure [17,18]. Seizure induction in the PTZ test was obtained by the i.p. injection of a convulsing dose of PTZ (100 mg/kg). Tonic-clonic convulsions, Seizures, hypnosis, and death were recorded. The highest active compounds **5b**, **5c**, and **5d** were further evaluated by using different doses in the PTZ model in order to determine their protective and therapeutic indexes. Three groups of six mice were used. Each group was given a range of i.p. doses of the test drug until at least four points were established in the range of 10%–90% from seizure protection or minimal observed neurotoxicity. The value of (ED_50_), slopes and standard errors were determined by using a computer program based on the method described by Finney [19,20].

### 3.9. Neurotoxicity Screening

Neurological toxicity (NT) was determined by a rotorod test in mice [14,15]. Groups of animals (mice) were trained to balance on a revolving rod (3 cm diameter and 6 rpm speed). The mice were allowed three trials to remain on the revolving rod for 20 s. The trained mice were given the test compounds i.p. at different dose levels. When the trained animals show lack of rolling roller performance then the test compound was considered neurotoxic. The taught animals were examined at 0.5 h and 4 h after the drug administration and then the neurotoxic symptoms were recorded as the median toxic dose (TD_50_). The acute neurotoxicity was tested following the method described by Dunham [14].

### 3.10. GABA Assay 

This assay was accomplished in brain tissue extracts enzymatically. Adult Wistar rats were separated into three groups of six animals each. After 2 h of i.p. drug administration (100 mg/kg), the rats were decapitated and the brains were put into separate vials containing 4–6 mL of ice-cold 80% ethanol and processed further following the reported procedures [27]. The chronic study was also performed through oral administration of the test compounds (30 mg/kg) for seven days. The data of GABA level concentration (μgm/100 mg of brain tissue) was collected as the minimum concentration that caused at least 50% inhibition established at one or more time points. 

## 4. Conclusions

Some novel derivatives of *N*-substituted-6-fluoro-quinazoline-4-amine were designed, synthesized, and biologically evaluated as anticonvulsant agents by using a subcutaneous pentylenetetrazole (scPTZ) test and a maximal electroshock (MES) test. Their neurotoxicity was also tested by using a rotorod test. All of the derivatives induced significant anticonvulsant activity. Three compounds **5b**, **5c**, and **5d**, showed the highest anticonvulsant activities in experimental mice with ED50 of 152, 165, and 140 mg/kg, while the reference drugs methaqualone and valproate had ED_50_ of 200 and 300 mg/kg. Structure activity relationship (SAR) studies explained that different substitutions on the phenyl ring of the aromatic amine exerted varied anticonvulsant activity. Changing the electronic nature and the position of the substituent group attached to the aromatic ring led to changing anticonvulsant activity. The three most active compounds had electron withdrawing groups at the 4-position of the phenyl ring. A 4-bromo substitution in **5d** showed higher activity than 4-chloro in **5b,** and this showed higher activity than a 4-fluoro in **5c**. These three compounds also showed a high safety margin and low neurotoxicity compared to the reference drugs. The molecular docking study was performed to evaluate the binding mode of the proposed compounds with the GABA-A receptor. The resulting data from the docking studies explained that all of the newly-synthesized derivatives had significantly high affinity towards the GABA-A receptor compared to methaqualone as a reference ligand. The data obtained from the biological screening integrated with that obtained from the modelling study. The enzymatic assay proved that these highly active compounds significantly increased the GABA level. The most active compounds **5b**–**d** could be used as a pattern for future design, optimization, and investigation to produce more effective analogs. 

## Figures and Tables

**Table 1:** Preliminary anticonvulsant screening of the newly-synthesized compounds in comparing with standard compounds methaqualone (0.5 mmol/kg) and valproate (1.8 mmol/kg).

Compound	PTZ (% Protection)	MES (% Protection)
**5a**	83	50
**5b**	100	100
**5c**	100	100
**5d**	100	100
**5e**	100	83
**5f**	83	66
**5g**	66	50
**5h**	66	33
**5i**	33	33
**5j**	50	33
Methaqualone	100	100
Valproate	100	100

**Table 2:** ED_50_, TD_50_, LD_50_, therapeutic index (TI), and protective index (PI) for the highest active compounds **5b**, **5c**, and **5d** compared to the reference drugs methaqualone and valproate.

Compound	ED_50_ (mg/kg)	TD_50_ (mg/kg)	LD_50_ (mg/kg)	Therapeutic Index (TI)	Protective Index (PI)
**5b**	152	270	580	3.82	1.78
**5c**	165	350	430	2.61	2.12
**5d**	140	320	550	3.93	2.29
Methaqualone	200	400	500	2.5	2
Valproate	300	450	500	1.67	1.5

**Table 3:** The calculated ∆G (free energy of binding) and binding affinities for the ligands.

Compound	∆G (kcal·mol^−1^)
**5a**	–55.74
**5b**	–68.97
**5c**	–67.50
**5d**	–69.68
**5e**	–66.84
**5f**	–58.81
**5g**	–62.65
**5h**	–65.68
**5i**	–56.04
**5j**	–65.76
Methaqualone	–50.69

**Table 4:** The values of GABA level in different areas of whole rat brain.

GABA con. (μgm/100mg of Brain Tissue)		
Compound	2 h Post-Treatment (100 mg/kg)	Seven Days Post-Treatment (30 mg/kg)
Control	45.8 ± 5.7	52.6 ± 3.4
**5b**	84 ± 4.42 **	79 ± 4.21 ***
**5c**	76 ± 5.28 **	80 ± 4.26 ***
**5d**	93 ± 8.42 *	105 ± 3.1
Clobazam	103 ± 5.9	109 ± 5.1

Values are represented as the mean ± SEM of six rats, significantly different from the control at * *p* < 0.001; ** *p* < 0.004; *** *p* < 0.01; (Student’s *t*-test). Reference drug clobazam was administered at 30 mg/kg [16].
